# Impact of COVID-19 on essential healthcare services in Addis Ababa, Ethiopia: Implications for future pandemics

**DOI:** 10.1371/journal.pone.0308861

**Published:** 2024-10-30

**Authors:** Admas Abera, Esete Habtemariam Fenta, Berhan Tassew Woldehanna, Firmaye Bogale Wolde, Meseret legesse, Lemma Demissie Regassa, Siobhan Mor, Mirgissa Kaba

**Affiliations:** 1 School of Public Health, Haramaya University, Harar, Ethiopia; 2 School of Public Health, Addis Ababa University, Addis Ababa, Ethiopia; 3 Knowledge Translation Directorate, Ethiopian Public Health Institute, Addis Ababa, Ethiopia; 4 Institute of Infection, Veterinary and Ecological Sciences, University of Liverpool, Liverpool, United Kingdom; 5 International Livestock Research Institute, Addis Ababa, Ethiopia; MeU: Mattu University, ETHIOPIA

## Abstract

**Background:**

Responding to the COVID-19 pandemic has presented an unprecedented challenge to health systems, with countries needing to balance the demands of responding directly to the pandemic, while simultaneously continuing provision of essential health services. This study aimed to explore the impact of COVID-19 on essential healthcare services in Addis Ababa, Ethiopia.

**Methods:**

A facility-based retrospective study was undertaken in 30 health centers in Addis Ababa which were selected using simple random sampling. Secondary data were extracted for 22 indicators on maternal and child health, communicable and non-communicable diseases, and outpatient services for the period spanning between July 2019 and October 2020. These indicators were selected based on the WHO operational guidance on maintaining essential health services during an outbreak guide, essential packages of health services in Ethiopia and expert consultation. The difference in the trends of services before and during COVID-19 was compared using linear-by-linear tests and the difference of magnitude across the indicators was compared using Autoregressive Integrated Moving Average (ARIMA) interrupted time series analysis at a 5% significance level.

**Results:**

Overall, more than 1.7 million people visited the studied facilities for outpatient services, and 18,325 mothers attended skilled delivery in the study period. The present study found that the mean number of patients treated for TB declined by 35 patients (β: -34.62; 95%CI: -50.29, -18.95) compared to the pre-COVID-19 era while the number of new patients enrolled for ART decreased by 71 patients (β: -70.62; 95%CI: -107.19, -34.05). Regarding maternal health services, the number of women who received post-natal care decreased by about 215 mothers (β: -214.87; 95%CI: -331.57, -98.17). Similarly, the mean number of clients served at inpatient services declined by 34 (β: -33.72; 95%CI: -68.55, 1.05). On the other hand, the mean number of patients screened for diabetes and hypertension during the pandemic increased by more than 1014 (β: 1014.5; 95%CI: 103.07, 1925.92) and 610 patients (β: 611.21; 95%CI: 302.42, 919.99), respectively. However, changes with regard to antenatal care, skilled birth delivery, and children immunization services did not show a statistically significant change after COVID-19 was reported in Ethiopia.

**Conclusion:**

Despite the notable efforts to sustain essential health services amidst the COVID-19 pandemic, our study revealed that there were disruptions in these services. This reinforces the need to adapt strategies to ensure sustainable provision of essential health services when pandemics of COVID19 magnitude cause disruptions of the health services.

## Introduction

The WHO’s declaration of COVID-19 as a pandemic in March 2020 highlighted the urgent need for global coordination [1]. Recognizing the potential disruption to essential health services, they swiftly released operational guidance in June 2020 with recommendations for practical actions that countries could take at national, sub regional and local levels [2]. Subsequently, in August 2020 they published the results of the first “pulse survey” which revealed that essential services in nearly all countries had been disrupted, particularly in low- and middle-income countries (LMICs) [3]. The second round of the “pulse survey”revealed that, more than one year into the COVID-19 pandemic, about 90% of countries were still reporting one or more disruptions to these health services [3, 4].

In addition to the poor infrastructure and inadequate human resource in LMICs, the health system lags behind high-income countries in terms of preparedness and resource allocation for essential health services [5]. When health systems are overwhelmed, direct mortality associated with disease outbreaks as well as other treatable conditions increases dramatically, requiring mobilization of resources for maintaining the health system [6]. Experience from the Ebola outbreak in West Africa revealed that the outbreak not only lead to a diversion of resources and cessation of health services, but also significant disruptions to antenatal and postnatal cares [7], immunization services [8], as well as Tuberculosis (TB) and Human Immunodeficiency Virus (HIV) [9] services. Similarly during COVID-19, it is estimated that 23 million children missed out on basic vaccines through routine immunization in 2020, which is 3.7 million more children compared to 2019 [10]. Thus, when the health system and health workforce are burdened due to the response to COVID19 [11, 12], countries need to balance the demands of responding directly to the pandemic, while simultaneously mitigating the risk of health system collapse.

Ethiopia is working to ensure equal access to essential health services, stating this as one of the major pillars in the country’s health sector transformation plan [13]. After the emergence of the pandemic, Ethiopia adapted the WHO guidance for maintaining provision of essential health services during COVID-19. The extent to which the country was able to achieve this objective has not been thoroughly assessed. To date, available literature on the effect of COVID-19 on essential health services focused on a single health facility [14], a specific service area [15] or used relatively short period of time [16]. Hence, the aim of this study was to investigate the impact of COVID-19 on essential healthcare services in Addis Ababa using routinely collected data for a period spanning before and during the pandemic (July 2019 to October 2020).

## Methods and materials

### Study design and setting

A retrospective study was conducted between February 15 and March 05, 2021 using patient registration data of health facilities in Addis Ababa. Hosting 30 percent of the urban population of Ethiopia, Addis Ababa had the highest number of COVID-19 cases in Ethiopia. The city is composed of three administrative levels: city administration at the top, 10 sub-cities, and 126 woredas. There are a total of 12 public hospitals, 99 health centers, and more than 400 various level private clinics in the city. Health centers are designated as the first tier in the three tier healthcare system which are primary healthcare units that provide services for an average of 40,000 individuals in urban areas [17].

### Inclusion/exclusion criteria

All public health centers providing essential and routine healthcare services in Addis Ababa and which report data to the Ministry of Health were eligible for inclusion. Hospitals because of the challenges to access patient registers and facilities dedicated to treating COVID-19 patients were excluded from this study since they did not provide essential health services. Private facilities were not included in the study as they do not report data to ministry of health. Hospitals.

### Sample size and sampling procedure

This study was conducted in 30 of the 99 health centers located in the 10 sub-cities of Addis Ababa (i.e. approximately 30% of the total health centers in the city). To identify the study sample, we prepared a sampling frame with the list of all health centers found in the 10 sub-cities of Addis Ababa. In each sub-city, health centers were divided based on performance in which they are classified into high, medium and low performers based on sets of criteria including the human resource they have, the number of people they serve and the type of service they provide. One health center was selected from each category using simple random sampling, for a total of 3 health centers from each of the 10 sub-cities. This ensured that all types of facilities from each sub-city were included in the study. The ranking of facilities was obtained from the respective sub-city health offices.

### Data extraction and indicators

We extracted data on the number of people attending essential health services from patient registries for a period spanning July 2019 to October 2020, that is for a total of 16 months (8 months before and 8 months after COVID-19 was reported in Ethiopia). Case data on maternal and child health (antenatal care visits (ANC) 1 and 4, skilled birth attendance (SBA), early postnatal care (PNC)), TB (all forms of TB cases detected, TB loss-to-follow up), HIV/AIDS (number of people on Anti-retroviral therapy (ART), newly started on ART, ART loss-to-follow up) and non-communicable disease (NCD) (cases of hypertension and diabetes mellitus), and in-patient admissions (which includes all types of patients admitted for any health problem) were extracted from the registries of each health facilities. Whereas data on total number of outpatient visits both for adults and children were extracted from the district health information system (DHIS2). In total, data on 22 healthcare indicators were extracted. These indicators were selected using the WHO operational guidance on maintaining essential health services during an outbreak guide [18], essential packages of health services in Ethiopia [19] and expert consultation. For this research purpose, data were extracted from health facilities from 15/02/2021 to 05/03/2021.

### Data analysis

Data were entered into excel and coded, cleaned and analyzed using STATA version 16. The data were analyzed separately by service areas, namely: non-communicable diseases, TB/HIV, maternal health, child health, inpatient admissions and outpatient visits. The monthly counts, means, and standard deviation (SD) were described for all indicators in the pre- and intra-COVID-19 periods. Differences in means for essential services were tested using linear-by-linear nonparametric tests at confidence level of 95% and p value ≤0.05. To determine the effect of COVID-19 on essential health services, we fitted binary Autoregressive Integrated Moving Average (ARIMA) model for each service. Type of ARIMA was determined by Autocorrelation and Cumulative periodogram white-noise test (Bartlett’s periodogram test). As all services have p-value of greater than 0.05, we considered white noise model of ARIMA with the following assumptions: residuals are white noise (there is no serial correlation, residuals are homoscedastic and mean of the residual is zero), and they are independent and stationary. Hence the ARIMA formula can be written as follows;

$$(1-\phi 1B-\cdots -\phi pB^{p})(1-B)dyt=c+(1+\theta 1B+\cdots +\theta qB^{q})\varepsilon t$$

Where:

1. (1−φ1B−⋯−φpBp) is Autoregressive (p)
2. (1−B)dyt is differences (d)
3. c+(1+θ1B+⋯+θqBq)εt is Moving Average (q)
4. y is each essential health services. As a result, we had 20 essential services and we estimated one ARIMA for each service which will totally be 20 coefficients.
5. y′t = (1−B)dyt′ = (1−B)dyt and μ is the mean of y′t
6. Assuming If c = 0 and d = 0d = 0, the long-term forecasts will go to zero. For d = 0, the long-term forecast standard deviation will go to the standard deviation of the historical data, so the prediction intervals will all be essentially the same.

### Ethics approval and consent to participate

Ethical clearances were obtained from Addis Ababa University College of Health Sciences Institutional Review Board (reference 070/20/SPH) and Central University Research Ethics Committees at the University of Liverpool (reference 8049). Due to the nature of the retrospective retrieval of patient data, the institutional review board of Addis Ababa University waived the requirement for an informed written consent. Permission was obtained from the Ministry of Health, Addis Ababa Health Bureau and each sub-city health offices to access required data. Before data collection, attempts were made to anonymize data by omitting names, card numbers, and any other identifying information to ensure patient privacy and confidentiality. Moreover, we only extracted the count of the required data elements and no other identifying information were collected.

## Results

Table 1 shows the total number of registered cases, and the monthly mean counts before and during COVID-19 for each indicator for the entire period under study (16 months). Overall, more than 1.7 million people visited facilities for outpatient services, about 45,000 mothers visited health institutions for the first antenatal care, and 18,325 mothers attended skilled delivery (Table 1). In addition, there were more than 50,000 hypertension cases, about 2,253 people started ART, 2,577 new TB cases were detected, and 41,445 under five children received pentavalent-3 vaccination.

**Table 1:** Summary statistics of essential health services utilization from July 2019 to October 2020 in Addis Ababa, Ethiopia.

Service areas	Total number of registered cases	Overall monthly mean (SD)	Before COVID-19, Mean (SD)	During COVID 19, Mean (SD)
**Outpatient and Inpatient services**				
Adult outpatient visits	1,409,440	88090 (-12487.6)	91852.25 (-5422.83)	84327.75 (-16504.4)
Under-5 outpatient visits	322,296	20143.5 (-4864.69)	23381.38 (-1367.24)	16905.63 (-4987.86)
Inpatient admission	1,788	111.75 (-34.47)	128.63 (-22.15)	94.88 (-37.48)
**Non-Communicable disease services**				
Diabetes cases	26,691	1668.19 (-321.56)	1680.25 (-386.95)	1656.13 (-267.43)
Hypertension cases	51,642	3227.63 (-636.96)	3310 (-768.37)	3145.25 (-513.32)
diabetes screened	29,122	1820.13 (-1085.37)	1312.88 (-930.94)	2327.38 (-1034.21)
Hypertension screened	109,922	6870.13 (-3452.29)	4766.88 (-3240.35)	8973.38 (-2219.93)
**TB and ART services**				
Number of people on ART	307,857	19241.06 (-606.49)	19034 (-425.97)	19448.13 (-713.27)
Newly started on ART	2,253	140.81 (-46.99)	176.13 (-34.4)	105.5 (-26.43)
ART Loss to follow-up	1,223	76.44 (-19.08)	87.38 (-8.05)	65.5 (-21.01)
Tuberculosis cases detected	2,577	161.06 (-24.18)	178.38 (-15.36)	143.75 (-18.2)
TB Loss to follow-up	66	4.13 (-4.65)	4.88 (-4.76)	3.38 (-4.72)
**Maternal health services**				
ANC-1	45,804	2862.75 (-324.79)	2896.75 (-235.63)	2828.75 (-409.74)
ANC-4	29,313	1832.06 (-151.84)	1854 (-185.65)	1810.13 (-117.63)
SBA	18,325	1145.31 (-84.37)	1135.38 (-100.57)	1155.25 (-70.09)
Early PNC	14,205	887.81 (-160.16)	995.25 (-117.75)	780.38 (-121.32)
**Child health services**				
PENTA-1	41,511	2594.44 (-211.51)	2540.38 (-229.65)	2648.5 (-190.91)
PENTA-3	41,445	2590.31 (-184.69)	2590.25 (-137.67)	2590.38 (-232.68)
Measles Vaccination	41,783	2611.44 (-621.89)	2555.88 (-176.96)	2667 (-889.03)
Full Immunization	38,186	2386.63 (-542.01)	2367.5 (-173.46)	2405.75 (-773.68)

### Non-communicable diseases

**Fig 1:**
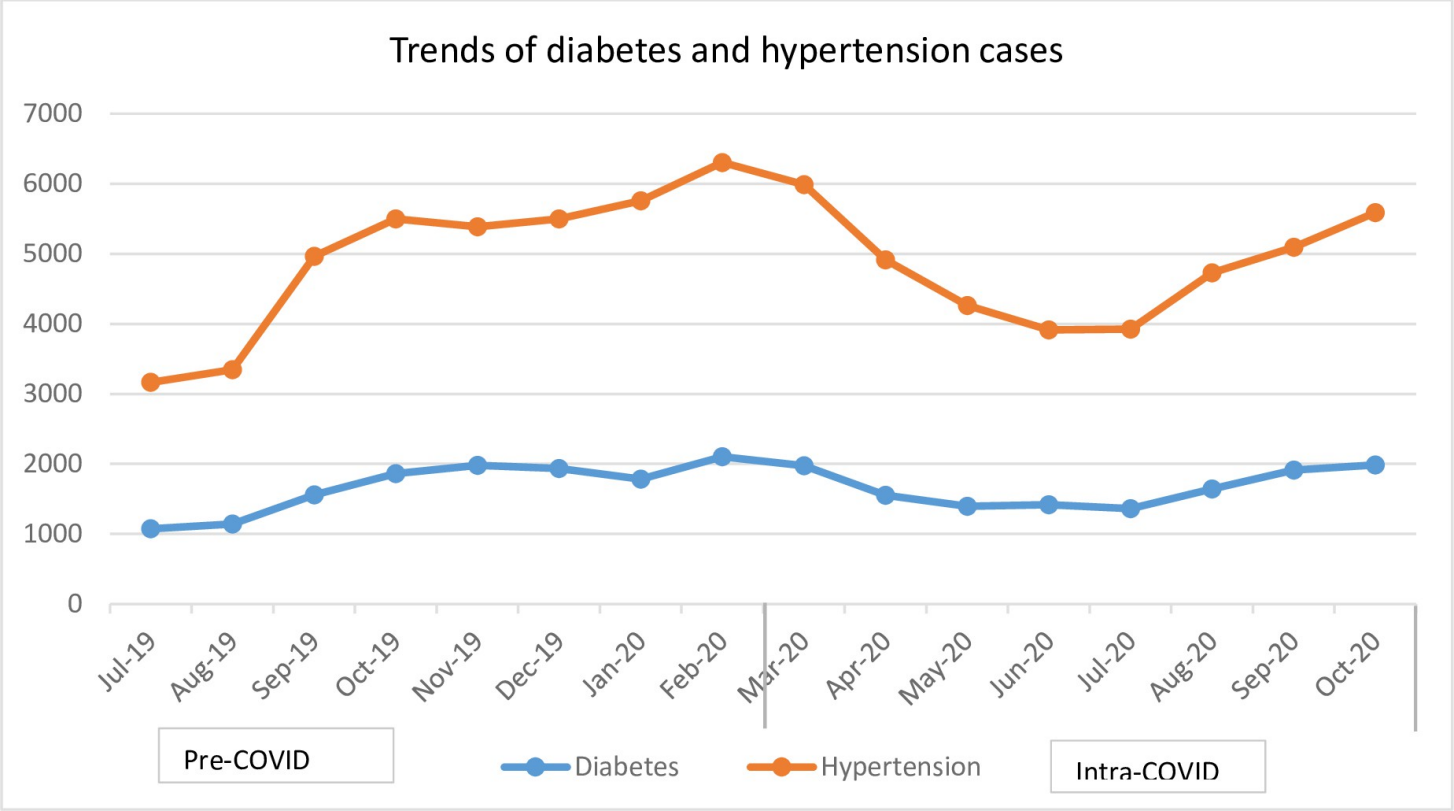
Trends of diabetes mellitus and hypertension; July 2019 to October 2020 in Addis Ababa Ethiopia.

Fig 1 shows the mean numbers of patients diagnosed with diabetes mellitus and hypertension in the study months. The mean number of patients screened for Diabetes was 1,312.87 (SD = 930.93) before COVID-19 and 2,327.37 (SD = 1034.21) during COVID-19 (Table 1). The monthly mean of screened patients for hypertension was 4,766.87 (SD = 3240.35) before and 8,973.37 (SD = 2219.92) during COVID-19, respectively. Overall, comparing the pre- and intra-pandemic periods, there was a 24.13 and 164.75 decrement in the mean number of detected diabetes and hypertension, respectively.

### TB and HIV services

**Fig 2:**
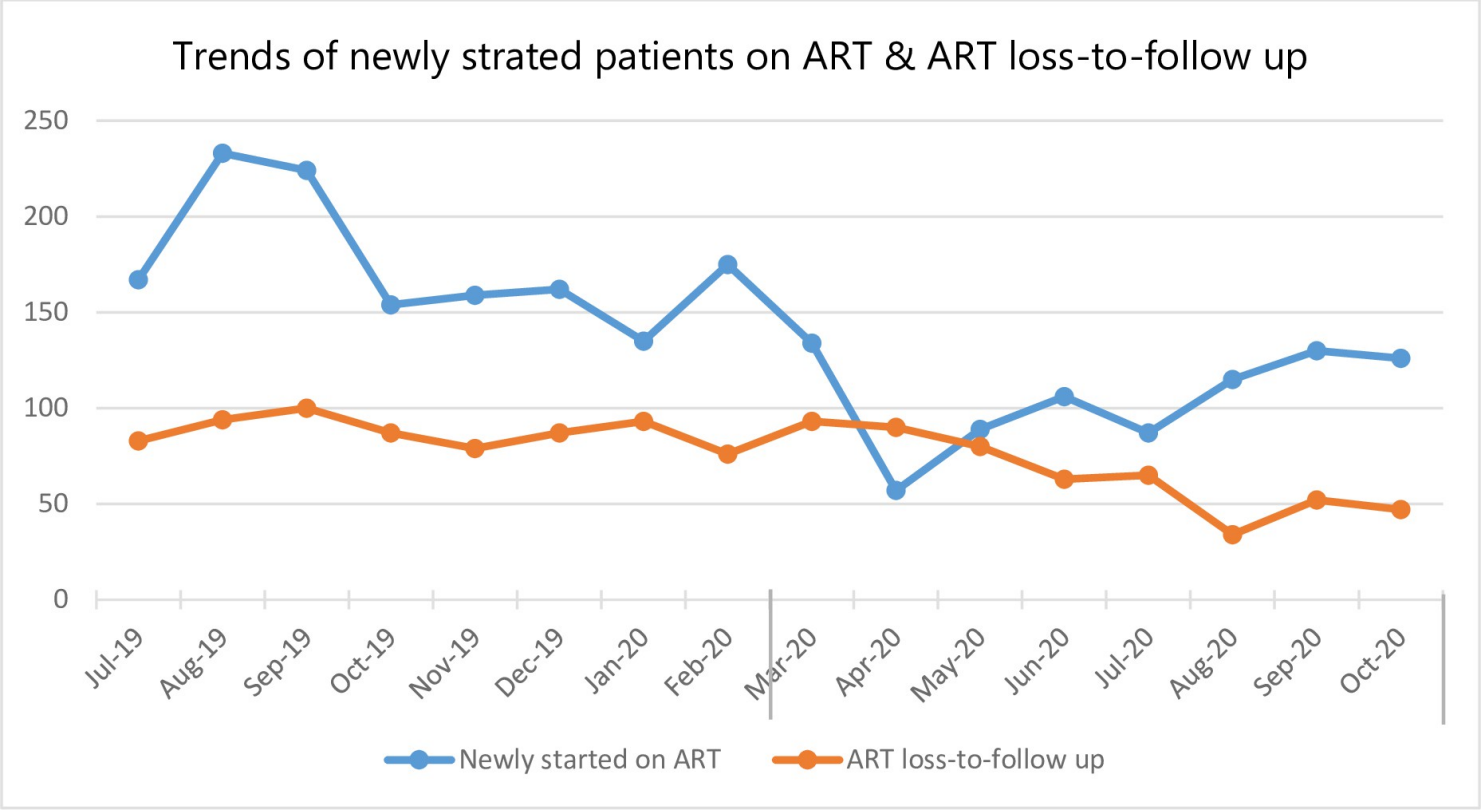
Trends of HIV related services in Addis Ababa Ethiopia from July 2019 to October 2020.

Overall, the mean number of people newly started on ART was 19,241.06 (SD = 151.62) and the average number of TB cases was 161.06 (SD = 6.043) (Table 1). The mean number of people on ART showed a decline from about 176.12 (SD = 12.16) during the pandemic to about 105.5 (SD = 9.34) before COVID-19. Meanwhile loss to follow up of patients on ART declined four months after first COVID-19 was reported in Ethiopia (Fig 2).

**Fig 3:**
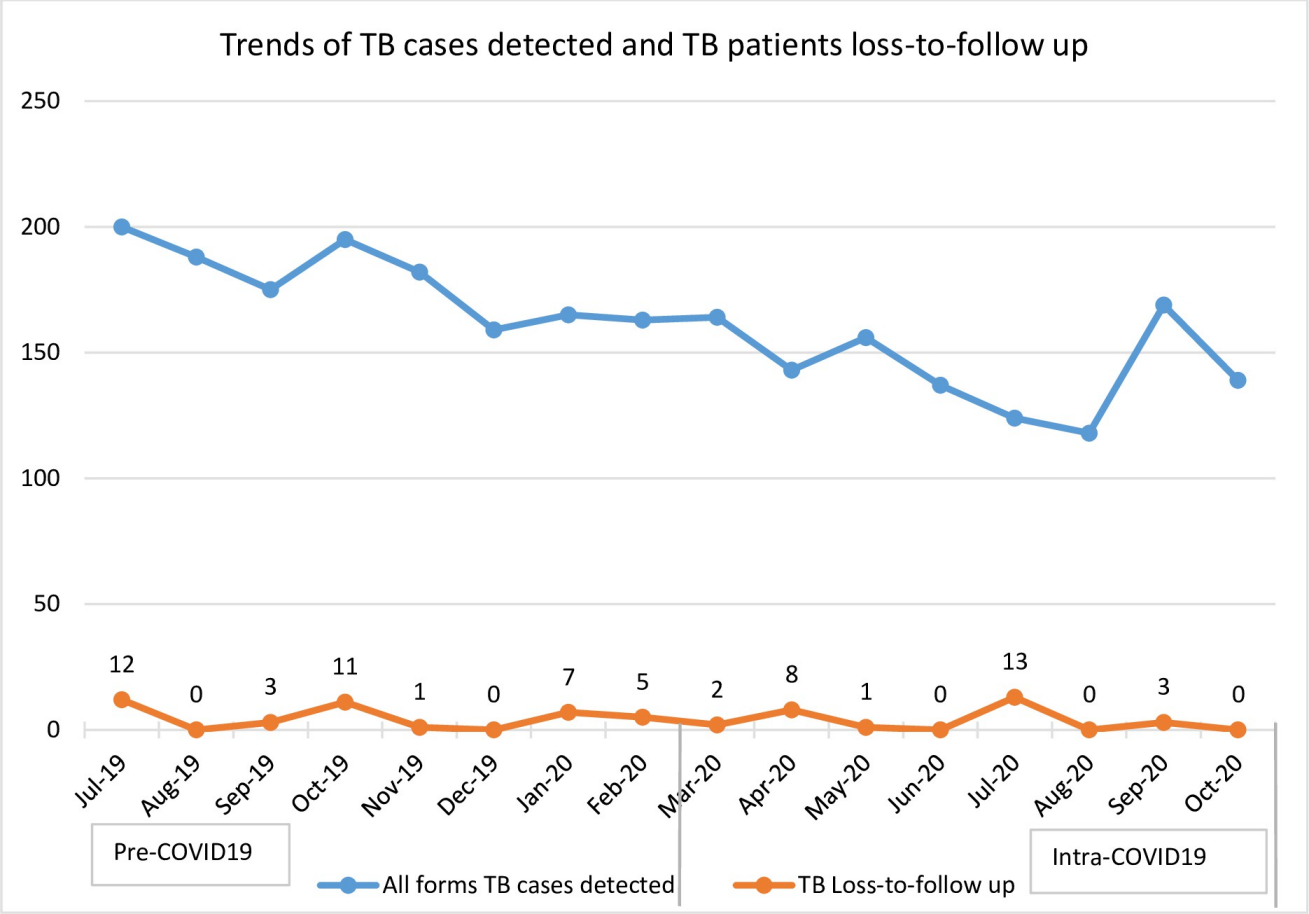
Trend of TB cases detected and TB loss-to-follow up in Addis Ababa Ethiopia from July 2019 to August 2020.

The number of TB cases detected declined from 178.37 (SD = 5.43) to 143.75 (SD = 6.43). During COVID-19, the first couple of months seen a drop in the number of patients newly started on ART and TB cases detected (Fig 3).

### Maternal health services

**Fig 4:**
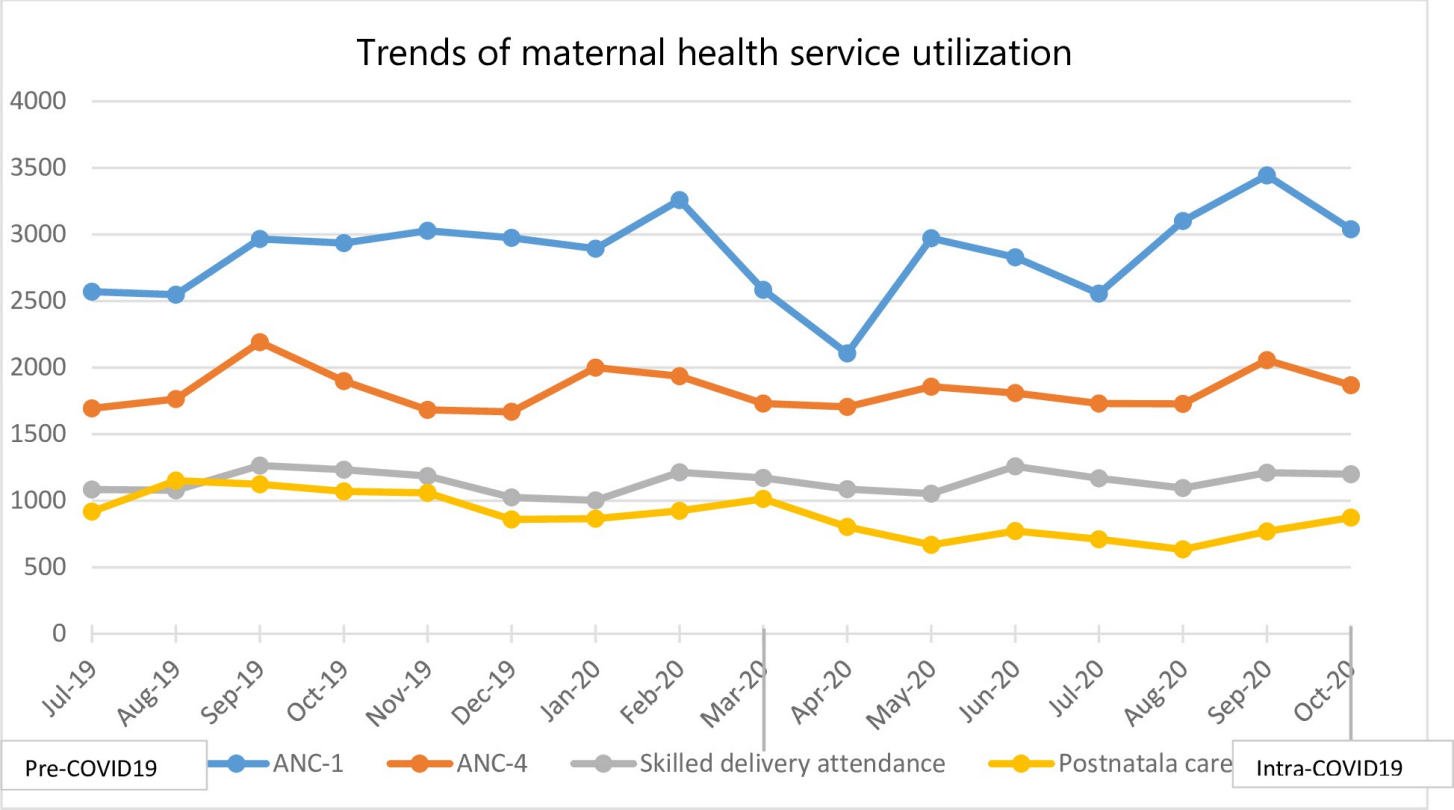
Trends of maternal health service utilization in Addis Ababa Ethiopia; July 2019 to October 2020.

After a more or less stable levels of maternal health service utilization before COVID-19 was reported in Ethiopia, early PNC and ANC-1 decreased steadily from February 2020 until April 2020 when it rose to a comparable level to the pre-COVID-19 period (Fig 4). The fourth antenatal visit and skilled delivery attendance remained relatively unchanged throughout the study period.

### Child health services

**Fig 5:**
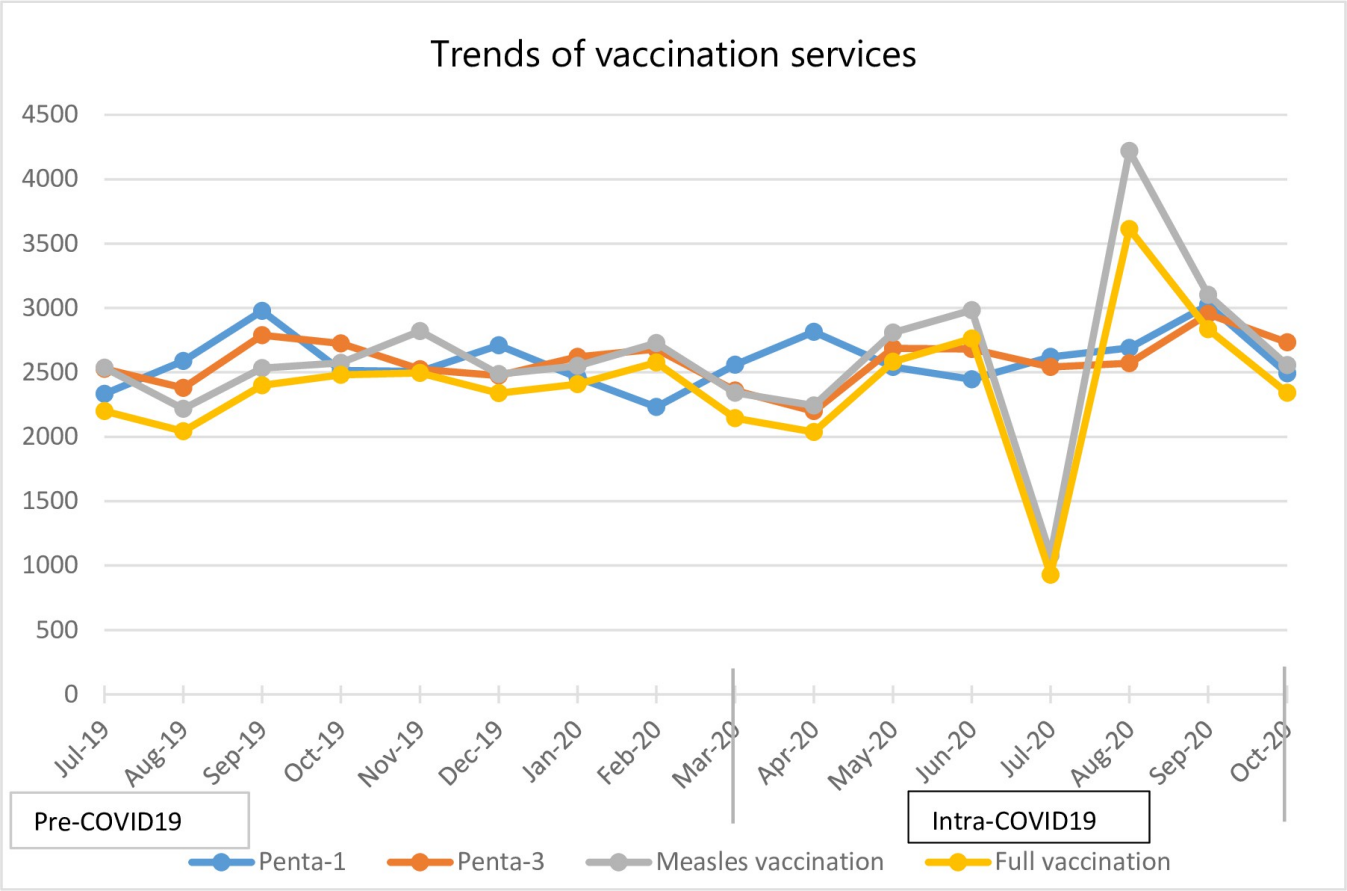
Trends of expanded immunization program in Addis Ababa Ethiopia from July 2019 to October 2020.

The number of children vaccinated against measles and fully vaccinated showed a sharp decrease in the month of July 2020 before it sharply rose in the following 3 months to reach pre-pandemic levels (Fig 5). This decrement was observed after 3 months of COVID-19 was first reported in the country.

### Outpatient and inpatient services

**Fig 6:**
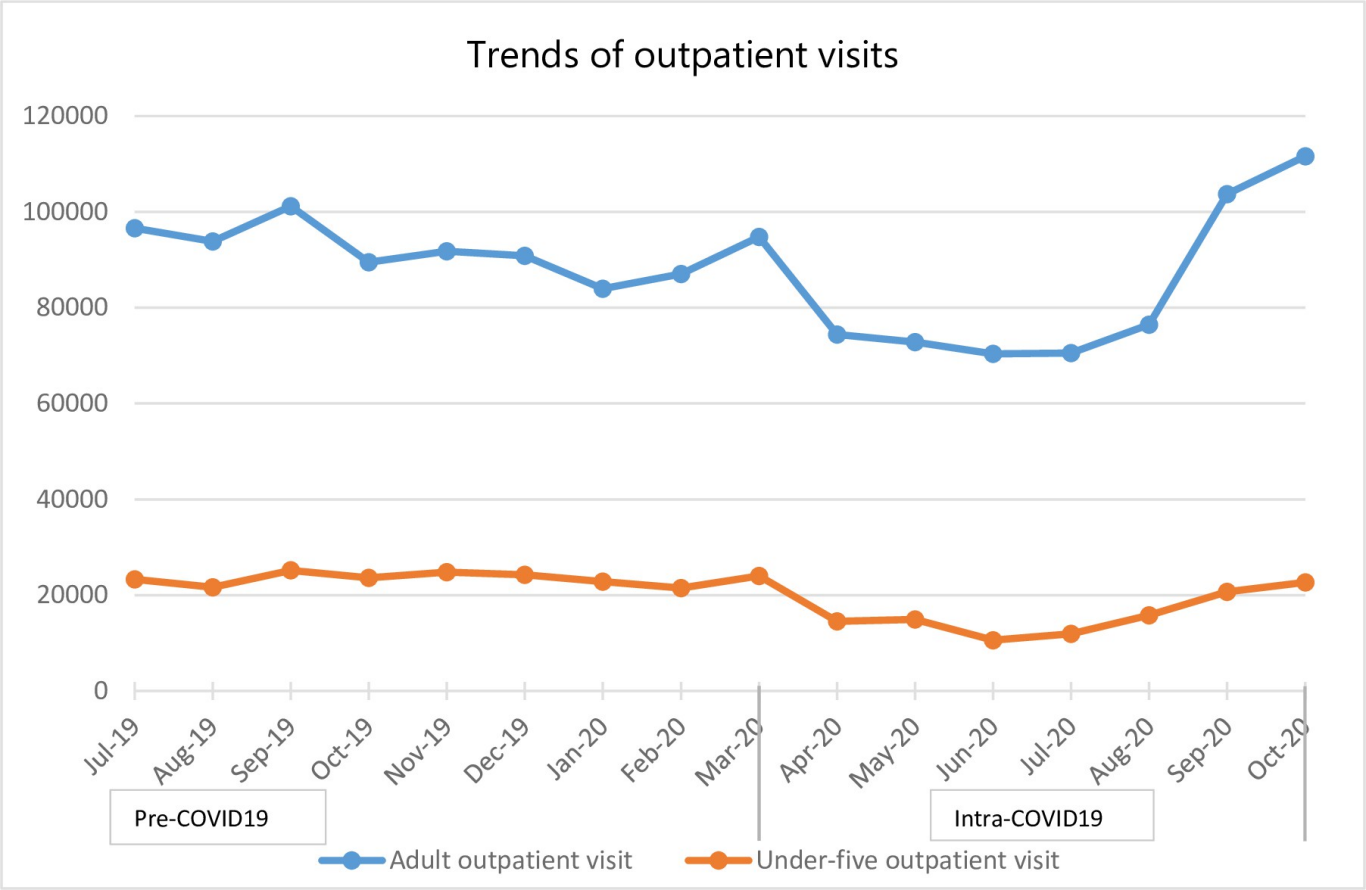
Trends in outpatient services utilizations in Addis Ababa Ethiopia from July 2019 to October 2020.

The number of people coming for both adult and child outpatient visits declined sharply in the month of March 2020 (the month COVID-19 was first detected in Ethiopia). It continued to decline for the following 5 months before it started to increase in August 2020 to reach pre-COVID-19 levels (Fig 6).

**Fig 7:**
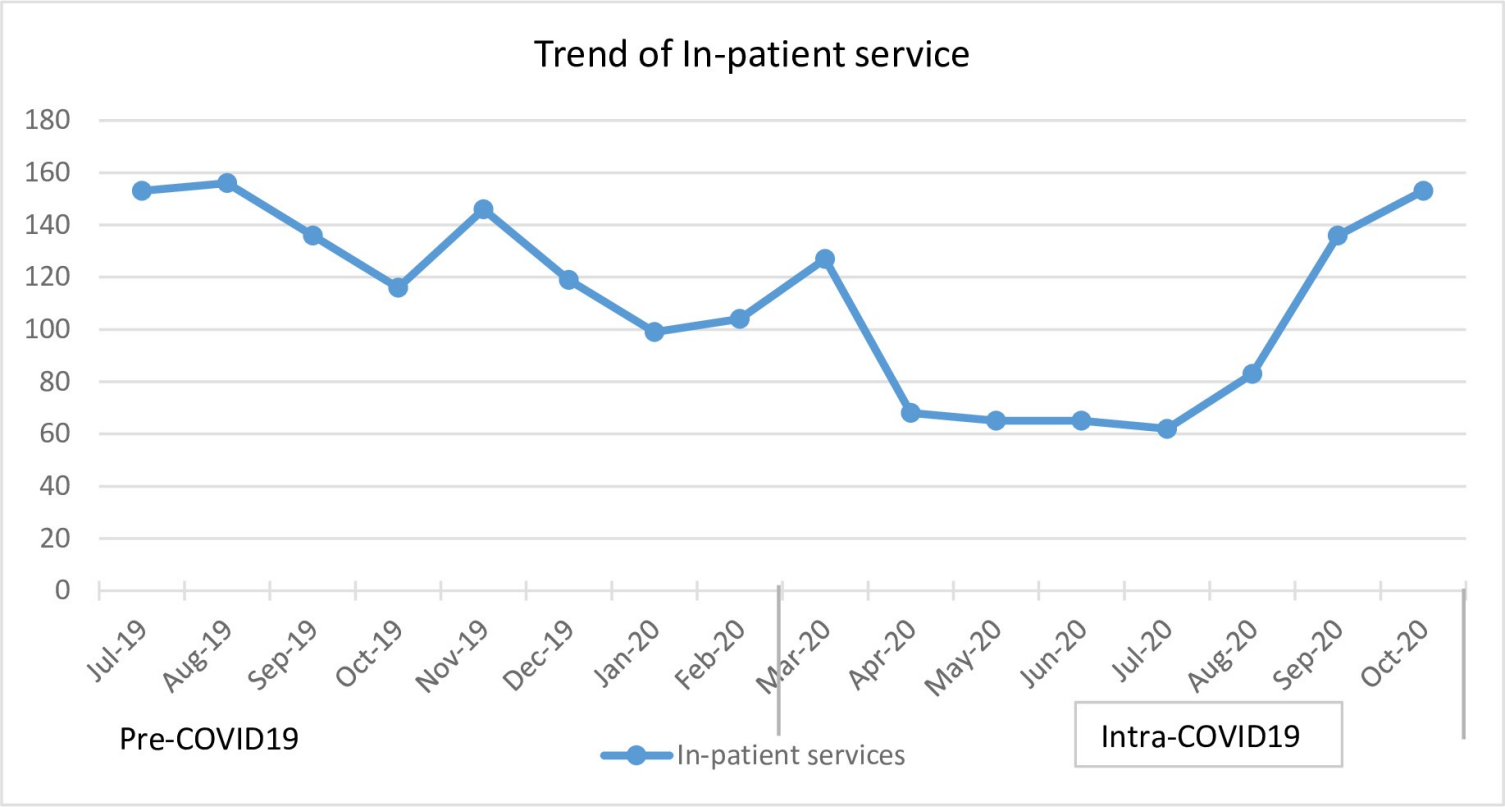
In-patient services utilizations in selected health facilities in Addis Ababa from July 2019 to October 2020.

Inpatient admissions showed a similar pattern with outpatient visits, steadily decreasing between April and August 2020 then starting to rise again in September 2020 (Fig 7).

### Trend analysis for essential health services over COVID-19 period

The difference in monthly trends of the selected essential services is summarized in Table 2.

**Table 2:** Trends of COVID-19 pandemic on essential health services in Addis Ababa, Ethiopia, from July 2019 to October 2020.

Variable (group)	Mean difference (before and during COVID-19)	Trend analysis linear-by-linear (Z (*p*-value))
**Outpatient and Inpatient services**		
Adult outpatient visit	-7,524.5	-1.25 (0.210)
Under five outpatient visits	**-6,475.75**	**-2.75 (0.006)**
Inpatient admission	**-33.75**	**-2.02(0.043)**
**Non-Communicable disease services**		
Diabetes cases	-24.13	-0.15 (0.870)
Hypertension cases	-164.75	-0.52 (0.605)
Diabetes screening	1,014.5	1.87(0.060)
Hypertension screening	**4,206.5**	**2.44 (0.014)**
**ART and TB treatment**		
People on ART	414.13	1.36(0.172)
People newly started on ART	**-70.62**	**-3.01 (0.002)**
HIV Loss-to-follow up	**-21.88**	-2.29 (0.022)
Total TB cases	**-34.63**	**-2.86 (0.004)**
TB Loss-to-follow up	-1.50	-0.65 (0.518)
**Maternal Health Services**		
ANC1	-68.00	-0.419(0.675)
ANC4	-43.88	-0.578(0.563)
SBA	19.88	0.471(0.637)
Early PNC	**-214.88**	**-2.683(0.007)**
**Child Health Services**		
PENTA-1	108.13	1.022(0.307)
PENTA-3	0.125	0.001(0.998)
Measles	111.125	0.357(0.721)
Full immunization	38.25	0.141(0.887)

The trend of patients treated at under five outpatient, and inpatient departments decreased after COVID-19 was detected in Ethiopia. The mean number of clients served at adult OPD decreased by 6476 while the inpatient service declined by 34. The mean number of patients screened for hypertension revealed an increment by 4207 (z = 2.44, p-value = 0.14). However, the cases of both hypertension and diabetes decreased despite the non-significant difference during COVID-19 periods. Regarding HIV and TB services, mean number of newly enrolled patients to ART treatment (z = -3.01, p-value = 0.002) and detection of TB (z = -286, p-value = 0.004) declined in the intra-pandemic compared to before COVID-19. The mean HIV treatment lost to follow up during COVID-19 have also decreased by about 35 patients (z = -2.29, p-value = 0.02). On the other hand, the monthly mean number of mothers who attend early PNC decreased by 215 patients in the study period (z = -2.683, p-value = 0.007).

### Effect of COVID-19 on selected essential health services

In our results, the p-values for the Ljung-Box chi-square statistics are all greater than 0.05. None of the correlations for the autocorrelation function of the residuals or the partial autocorrelation function of the residuals are significant. Hence the model meets the assumption that the residuals are independent. In the bivariate ARIMA model, the effect of COVID-19 was significantly recorded for screening for diabetes mellitus and hypertension, TB cases on treatment, newly initiated ART, lost to follow up of people on ART and early postnatal services. Both diabetes and hypertension screening services increased during COVID-19 period. To the contrary, other services including all form TB cases, newly initiated ART services, ART lost-to-follow up, and early post-natal care revealed a significant decrement during the pandemic.

More specifically, the monthly mean number of patients screened for diabetes mellitus during COVID-19 increased by more than 1,014 (β: 1014.5; 95%CI: 103.07, 1925.92) while hypertension screening increased by more than 600 people (β: 611.21; 95CI:302.42, 919.99). The present study also found that the mean number of patients treated for TB declined by 35 (β: -34.62; 95%CI: -50.29, -18.95) compared to the pre-COVID-19 era. The monthly mean number of new HIV patients enrolled for ART in the months of COVID-19 also decreased by more than 70 patients (β: -70.62; 95%CI: -107.19, -34.05) while the monthly mean of lost-to-follow-up of people on ART declined by 22 patients (β: -21.87; 95%CI: -43.70, -0.05). Regarding the maternal health services, our study indicated that the mean number of women who received early post-natal care decreased by 215 (β: -214.87; 95%CI: -331.57, -98.17). However, changes with regard to both the first and fourth antenatal care, skilled birth delivery, and vaccination services did not show a statistically significant change after COVID-19 was reported in Ethiopia (Table 3).

**Table 3:** ARIMA results to determine effects of COVID-19 pandemic on essential health services in Addis Ababa, Ethiopia. Negative and positive coefficients indicate decrease and increase in service use, respectively.

Effect of COVID 19	Coefficient (95% CI)	*p*-value	SE	Sigma
**Outpatient and inpatient services**				
Inpatient service	-33.75 (-68.55, 1.05)	0.057	17.76	28.795 (9.972, 47.62)
Adult OPD	-7524.5 (-26956.64, 11907.64)	0.448	9914.54	11490.82 (6293.58, 16688.07)
Under five OPD	-6475.75 (-13073.71, 122.208	0.054	3366.37	3420.86 (2137.07, 4704.65)
**Chronic Non-communicable disease services**				
Screened for Diabetes	1014.5 (103.07, 1925.92)	0.029	465.02	920.38 (482.27, 358.49)
Total Diabetes cases	-24.13 (-398.98, 350.74)	0.90	191.26	311.1184 (118.06, 504.17)
Hypertension screening	611.21 (302.42, 919.99)	<0.001	157.54	2598.03 (1066.58, 4129.46)
Total Hypertension cases	-164.75 (-892.38, 562.88)	0.657	371.24	611.2116 (302.43, 919.99)
**TB and HIV treatment services**				
Tuberculosis cases	-34.62 (-50.29, -18.95)	<0.001	7.99	15.75 (6.81, 24.69)
TB Loss-to-follow up	-1.5 (-6.47, 3.47)	0.554	2.53	4.43 (1.47, 7.40)
No. of people on ART	414.125 (-511.38, 1339.63)	0.380	472.21	549.51 (176.34, 922.69)
Newly initiated ART	-70.62 (-107.19, -34.05)	<0.001	18.66	28.69 (13.03, 44.36)
Loss-to-follow up of people on ART	-21.87 (-43.70, -0.05)	0.049	11.14	14.88 (9.37, 20.39)
**Maternal Health Services**				
ANC 1	-68 (-423.00, 287.00)	0.707	181.13	312.64 (203.96, 421.30)
ANC 4	-43.87 (-201.72, 113.97)	0.586	80.54	145.37 (80.84, 209.89)
Delivery service	19.87 (-64.82, 104.57)	0.646	43.21	81.08 (32.57, 129.59)
Early PNC	-214.87 (-331.57, -98.17)	<0.001	59.540	111.82 (51.58, 172.06)
**Child Health services**				
PENTA-1	108.12 (-89.33, 305.58)	0.283	100.74	197.53 (114.45, 280.62)
PENTA-3	0.125 (-200.59, 200.84)	0.999	102.41	178.83 (115.23, 242.42)
Measles 1	111.12 (-1423.33, 1645.58)	0.887	782.89	599.57 (467.12, 732.02)
Full immunization	38.25 (-1166.54, 1243.04)	0.06	614.70	524.45 (401.60, 647.28)

## Discussion

This study found that there were disruptions in essential health services during the pandemic. A significant decline in service utilization was recorded for TB and HIV treatments, and post-natal care while slight increase was observed for diabetes mellitus and hypertension screening services. Even though the change was statistically not significant, there was also a decrease in inpatient as well as under five and adult outpatient departments. Disruptions in general attendance for health services as a result of the pandemic were common findings reported in other studies [20–22]. As the COVID-19 pandemic continued and strict control measures including lockdowns and social distancing were introduced, disruption of essential preventive and curative health service was anticipated.

At the beginning of the pandemic in Ethiopia, most health facility visits were limited to emergency services and other severe medical conditions. Most routine healthcare appointments were either cancelled or postponed with the aim of reducing transmission of the virus. Prescription refill for a longer duration of time was practiced for vulnerable groups including for people with chronic diseases, including patients on anti-TB medications, and ART.

The present study found that outpatient visits for both adults and children decreased. During COVID-19, adult outpatient visit decreased by 10% when compared with the pre-COVID-19 times whereas under five outpatient visits decreased by almost one third. In addition, the proportion of outpatient visits for the top ten diseases significantly decreased by more than two thirds. Our findings align with the WHO pulse surveys on the continuity of essential health services conducted in 105 countries [3, 4], in which most low-income countries reported outpatient service disruptions due to the pandemic. Country-specific studies in Ethiopia [14], Kenya [23] and Uganda [24] also reported similar findings.

Consistent with previous studies [3, 25], our analysis showed a decrease in inpatient admission. This decrement may be attributed to the fear of acquiring the virus from both health care provider and the community, particularly in Ethiopia’s early months of COVID-19 diagnosis [26, 27]. Further, some health facilities were dedicated to providing COVID-19 related services, which might have contributed to this reduction.

Maternal and child health services are critical for women and children as disruption may lead to adverse outcomes, including unintended pregnancies and health risks for mothers and children [4]. Post-natal care during COVID-19 was found to be significantly decreased in this study. The decrement could have resulted from the practice of sending mothers home earlier (within 6 hours of delivery) to decrease the burden on the maternity ward and reduce exposure. Another reason that post-natal care decreased might be due to the increase of home delivery as the number of women who follow antenatal care decreased (>60 for ANC 1 and >28 for ANC 4). In contrast to previous studies [28, 29], our study found that other maternal services were not significantly affected due to COVID-19. According to the WHO pulse survey conducted in 2020, antenatal and delivery services were rarely severely disrupted [3]. In Ethiopia, amid the pandemic, most health facilities were providing and contacting mothers for antenatal and other maternal services through telephone. However, our finding were consistent with recently conducted studies elsewhere [25, 29].

Immunization services, essential to preventing several infectious diseases in children, were not negatively affected in our study. The number of children vaccinated for Pentavalent-1, Pentavalent-3, and measles vaccines and those fully vaccinated did not show statistically significant change during the COVID-19 period compared to the preceding months. The WHO pulse survey also reported that routine immunization services were disrupted in only 10% of the 105 studied countries [4]. Our finding was also consistent with other studies in LMICs [30–32].

Similarly, a large study [33] among 15 African nations showed that countries had maintained the cumulative monthly average vaccination, which corroborates our finding. The seemingly minimum disruption of immunization services may be due to the fact that the ministry of health and the health institutions were actively engaged with the community to reduce discontinuation of the service. In the study area, for instance, health facilities through a family health team were providing regular health education on the importance of continuing essential health services during the pandemic.

The number of patients screened for diabetes mellitus and hypertension dramatically increased in our study. Before COVID-19, both diabetes mellitus and hypertension were not given adequate attention and there was no non-communicable disease surveillance [34]. Further, as indicated in the study conducted by Helamo et al in 2017, the record of both services was poor as services were also not included in the Key Performance Indicator (KPI) of Ethiopia [35]. After the emergence of the pandemic, death and other complications of COVID-19 were worsened by comorbidities. As a result, the health system initiated the promotion of screening for chronic disease including hypertension and diabetes mellitus which in turn affected the flow of clients and records in health institutions. However, COVID-19 did not significantly impact total attendance for hypertension and diabetes patients, which was in line with other studies elsewhere [25, 36]. In contrast, the WHO essential health services continuity survey indicated that 45% and 42% of countries did report disruptions to hypertension and diabetes management services, respectively [1]. Another study indicated that the number of non-communicable disease services declined during the peak of the COVID-19 pandemic, before showing a limited recovery [37]. Although there were interventions to ensure the continuity of hypertensive and diabetes services, the slight reduction in non-communicable diseases may be due to the fear of acquiring COVID-19 and the reduced facility visit counselling for vulnerable groups.

Regarding infectious diseases, a decline was observed in the number of patients who were newly started on ART, as well as the number of TB cases detected. In one study in Kenya, the number of people with presumptive pulmonary TB showed an overall 31% decrease and a decline in the number HIV tested people by half [38]. A modelling study illustrated that the most significant impact observed was reductions in timely diagnosis and treatment of new cases of TB and while for HIV, it was from loss to follow-up [39]. Other studies also reported similar results [40–42].

The present study explored the burden posed by COVID-19 on essential health services, which covered a wide range of health needs of different age groups and related to Ethiopia’s most important sources of morbidity and mortality. Hence, these findings can be of considerable use for health system leaders and policy makers to respond to essential health services during pandemics. Quantitative analysis of changes in health service utilization is prone to biases due to data quality issues, lack of a control group to attribute the observed difference and the negative influence of COVID-19 on reporting. However, extracting data directly from the patient registry books greatly reduces the risk of missing reports. It is important to note that the study did not include private facilities and hospitals. Therefore, the findings may not be generalizable to the entire healthcare system in Ethiopia.

## Conclusions

Despite the notable efforts to sustain essential health services amidst the COVID-19 pandemic in Ethiopia, the present study found that there were disruptions in these services. Outpatient visits for both adults and children, and in-patient admissions were disrupted as a result of the pandemic. Moreover, a decline in numbers was significantly recorded for TB, HIV treatment, and postnatal care whereas immunization services were not significantly affected. In order to mitigate the impact, health system actors have made concerted efforts to respond to the pandemic while sustaining provision of essential healthcare services. It is reasonable to assume that even a modest disruptions in essential services could lead to increased morbidity and mortality from causes other than the COVID-19 or other pandemics in the short, medium or long term. This reinforces the need to adapt strategies to ensure sustainable provision of essential health services. The Ministry of Health, local health bureaus and stakeholders should monitor changes in service delivery and utilization to balance the response to future pandemics and sustain provision of essential health services.

## Supporting information

S1 File Maintaining essential health services during COVID19. (DOCX)

S2 File COVID19 effect on essential services. (XLS)

## List of abbreviations

ANC: Antenatal Care
ARIMA: Autoregressive Integrated Moving Average
ART: Anti-retroviral Therapy
BSRC: Biological Sciences Research Council
DHIS: District Health Information System
GCRF: Global Challenges Research Fund
HIV: Human Immunodeficiency Virus
HORN: One Health Regional Network
KPI: Key Performance Indicator
LMICs: Low- and Middle-income Countries
NCD: Non-communicable Disease
OPD: Outpatient Department
PNC: Postnatal Care
SBA: Skilled Birth Attendance
TB: Tuberculosis
UKRI: UK Research and Innovation
