# Supplementary material for: Impact of COVID-19 on essential healthcare services in Addis Ababa, Ethiopia: Implications for future pandemics
Source: PLoS One. 2024 Oct 30;19(10):e0308861. doi: 10.1371/journal.pone.0308861 (PMC11524496; doi:10.1371/journal.pone.0308861)
Supplement: S1 File — (DOCX) [file pone.0308861.s001.docx]

**Maintaining essential healthcare services in Addis Ababa during COVID-19: A qualitative study**

Esete Habtemariam Fenta^1^, Berhan Tassew^*1^, Admas Abera^2,^ Firmaye Bogale^3^, Meseret legesse^2^, Justin Pulford^4^, Siobhan M. Mor^5,6^ and Mirgissa Kaba^1^

^1^School of Public Health, Addis Ababa University, Addis Ababa, Ethiopia

^2^Epidemiology and Biostatistics Unit, School of Public Health, Haramaya University, Harar, Ethiopia.

^3^Knowledge Translation Directorate, Ethiopian Public Health Institute, Addis Ababa, Ethiopia.

^4^Department of International Public Health, Liverpool School of Tropical Medicine, Liverpool, UK

^5^Institute of Infection, Veterinary and Ecological Sciences, University of Liverpool, Liverpool, UK

^6^International Livestock Research Institute, Addis Ababa, Ethiopia

**^*^Corresponding Author**

Berhan Tassew

School of Public Health, Addis Ababa University

Email: [tassewberhan@gmail.com](mailto:tassewberhan@gmail.com)

**Abstract**

**Introduction:** Worldwide, health systems have been challenged by the overwhelming demands of the COVID-19 pandemic. In Ethiopia, maintaining essential health services during the COVID-19 pandemic is critical to preventing severe outcomes and protecting the gains made over the past years in the health sector. This project aimed to explore the health system’s response to maintaining essential healthcare services in Addis Ababa, Ethiopia.

**Methods:** A total of 60 key informant interviews were conducted by purposively selecting key stakeholders from Federal Ministry of Health, Addis Ababa Regional Health Bureau, Sub-city Health Offices and frontline healthcare providers. Interviews were transcribed verbatim and coded using Open Code. Thematic analysis was employed to analyze the data.

**Result:** COVID-19 affected delivery of essential health service in several ways namely: decline in health service utilization, fear of infection among healthcare providers, stigma towards healthcare providers and perceived decrease in quality-of-service provision. However, the health system actors made efforts to sustain services while responding to the pandemic by enacting changes in the service delivery modality. The most significant changes in the service delivery included repurposing of health centers and prolonged prescription. The primary challenges encountered were burnout of health workforce and shortage of personal protective equipment.

**Conclusion:** COVID-19 has affected delivery of essential health service in multifaceted ways. System actors have accordingly made efforts to sustain services while responding to the pandemic.

**Key words: COVID-19, mitigation strategy, essential healthcare service, Ethiopia**

**Background**

On March 13, 2020, a COVID-19 positive case was first reported in Ethiopia. By March 28, 2021, a total of 200,563 confirmed COVID-19 cases and 2801 deaths were recorded in the country (1). Globally, health systems have been challenged by the overwhelming demands of the COVID-19 pandemic (2–9). Responding to the healthcare burden imposed by the pandemic required intense resources as this had to be coupled with ensuring continued access to essential healthcare services (10). Countries struggled to allocate resources for these needs because health facilities operated with less capacity due to the distribution of resources including health care providers for COVID-19 prevention, detection, and case management (11,12). Moreover, infection among health care providers resulted in missed working days and backlog of care (13,14).

Worldwide, almost every country (90%) experienced disruption to its health service, but low-and middle-income countries reported greater difficulties (15,16). The most frequently (more than 50%) disrupted essential health services were routine immunization services, outreach services and facility-based services, noncommunicable disease diagnosis and treatment, family planning and contraception, treatment for mental health disorders, antenatal care and cancer diagnosis and treatment. The disruptions mainly occurred more at the earlier stage of the pandemic and reduced with time (17,18). In addition to mortality and morbidity directly attributed to COVID-19, the pandemic poses a significant risk of indirect morbidity and mortality from other preventable and treatable diseases if essential health services are disrupted, as might occur when available healthcare resources are diverted to the fight the pandemic (19).

The World Health Organization recommended ensuring continuation of essential services including vaccination, chronic disease follow-up and maternal and child health care, by taking the local context and extent of outbreak into consideration (20). Maintaining essential health services during the COVID-19 pandemic is critical to prevent severe outcomes and protect the gains made over the past years in the health sector. In recognition of the burden of the pandemic in the health sector, the Ethiopian Federal Ministry of Health (FMOH) developed national guidelines for managing COVID-19 in April 2020. The guideline set standards for surveillance, tracing protocols, COVID-19 treatment centers, as well as health facilities preparedness, community engagement, and maintaining essential services during the pandemic (21). The Ethiopian essential health service package includes reproductive, maternal, neonatal, child, and adolescent health services; major communicable diseases; non-communicable diseases; surgical care; and emergency and critical care (22).

The pandemic has exposed the limitations of many health systems, including some that have been previously classified as high performing and resilient (23). A comprehensive analysis of the resilience of health systems during the pandemic can therefore pinpoint important lessons and help strengthen countries’ preparedness, response, and approach to future health challenges (20). Therefore, it was the aim of this research to explore the health system response to maintaining essential healthcare services in Addis Ababa, Ethiopia.

**Methods**

**Study setting**

This qualitative study was conducted in the capital of Ethiopia, Addis Ababa, which has a population of 8,938,683 million (30% of the country’s urban population). At the time of the research, the city had the largest number of reported COVID-19 cases in Ethiopia.

Ethiopia has a three-tier healthcare delivery system: level one is a Woreda/District health system comprised of a primary hospital (to cover 60,000-100,000 people), health centers (1/15,000-25,000 population) and their satellite health posts (1/3,000-5,000 population) connected to each other by a referral system. The primary hospital, health center and health posts form a primary health care unit (PHCU). Level two is a general hospital covering a population of 1-1.5 million people; and level three is a specialized hospital covering a population of 3.5-5 million people (24).

**Study approach and period**

We conducted a qualitative study to explore the health system response to sustain provision of essential healthcare services. This study was conducted from February to March 2021.

**Sample size and study participants**

We conducted a total of 60 key informant interviews. We purposively selected participants from the FMOH, Addis Ababa regional health bureau, and Sub-city health office who were assigned to COVID-19 response team. In addition, we interviewed the medical director/focal point of the rapid response team and service providers at health centers from different contact points that provide essential health services. All participants selected for the interviews were those expected to be the most knowledgeable about the mitigation strategies and/ or the health system response to sustain provision of essential healthcare service in their respective department.

**Data collection**

Interview guides were developed with open-ended questions to ensure a high degree of flexibility. Interview guides covered topics such as the effect of COVID-19 on essential healthcare service provision and utilization, activities undertaken to sustain provision of essential healthcare service, infection prevention and control (IPC) measures taken to ensure safe delivery of essential healthcare service, and challenges faced by the health system while providing essential healthcare service during the pandemic. Interviews were arranged for a time and location that was deemed most favorable and comfortable for the participants. Interviews were conducted in a quite office and privacy was ensured to provide participants the freedom to express their opinions. All interviews were audio taped and took about an hour on average. All data were collected by five experienced and trained primary and co-primary investigators. During data collection, respondent validation was done by restating and paraphrasing information for respondents to determine the accuracy. Additionally, investigator triangulation was applied by using multiple investigators to study the same phenomena improving the credibility of findings of the study.

**Data analysis**

Thematic data analysis was used to describe and compare general statements as relationships and themes present on the data (25). The interviews were first transcribed verbatim into Amharic then translated into English. A codebook was developed based on initial review of the transcripts. Next, transcripts were systematically coded using open code (V 4.02). Double coding was initially used with coding disagreements resolved by discussion and updates to the code definitions where needed. Double coding proceeded until no new disagreements were identified. After this, the transcripts were single coded. Lastly, the codes were categorized into themes and compared and contrasted across each theme.

Rigor was enhanced through regular discussions between researchers who read all interview transcripts, counter checked the transcripts, coded the data, and agreed on the emerging themes after going through the data.

**Results**

**Theme 1: Effect of COVID-19**

**Effect on service utilization**

Participants explained COVID-19 resulted in an overall reduction in patient flow, loss-to-follow-up and defaulting on treatment. This was especially true in the first few months after COVID-19 was first reported in Ethiopia.

“*During COVID-19, clients did not want to come to HC at all because they thought they would get COVID in addition to their health problem. The patient flow was almost close to zero at one time. But once the effect of COVID decreased the panic among the public also started decreasing and patients started coming back to health center (HC). That’s when we saw patients who have stopped their medication.”*

Our finding indicated that clients in Antiretroviral Treatment (ART) clinics were missing their appointments causing clients to suffer from opportunistic infections.

*" ART clients did not come here as they used to since they are more susceptible to COVID-19 due to their immune status. There were also some clients who were lost-to-follow-up due to fear of contracting the infection by coming here.”*

Similarly, the number of patients with non-communicable disease (NCD) who came for follow-up significantly reduced due to fear of contracting COVID-19 at health facilities. Some patients were known to have discontinued their medication while others started buying medication from private pharmacies without follow-up. This caused patients with NCD to suffer from complications. A participant mentioned the situation as follows:

*“…we saw some patients who have stopped taking medication. The number of people with complications like diabetic ketoacidosis (DKA) and hypertensive emergency has also increased because of not having follow-up.”*

Our findings also indicated that patients with tuberculosis (TB) refused to come for follow up and even those coming to the health facilities did not want to stay long for physical examination and laboratory investigation. In addition, people who had cough were hiding their symptom and using home remedies hoping they would get better from fear of being diagnosed with COVID-19 and being quarantined. This led to late detection and low diagnosis of TB during the earlier phase of COVID-19 in the country.

Participants indicated that the number of visits for delivery, family planning and first antenatal care visit had shown a decrement. Participants mentioned that the number of unwanted pregnancy and abortion increased. Similarly, pregnant mothers delayed or missed their appointment to avoid exposure and there were home deliveries.

*“As a result of COVID-19 the number of mothers coming for follow up has decreased. There was interruption of Antenatal care (ANC) visits and we have also seen few home deliveries.”*

Participants mentioned that families were not willing to bring their children for immunization due to fear of infection at health facilities. In addition, some were reluctant to get their children vaccinated during campaigns for childhood diseases thinking it would be a trial for a COVID-19 vaccine. One participant explained this as follows:

*“At the beginning of the pandemic there were vaccination campaigns for measles and polio, and we have seen fear among the community. They did not believe we were providing polio, or measles vaccines. Thus, they refused to get their children vaccinated and said they do not want to vaccinate their children because of fear.”*

Our findings also indicated that health service utilization by people with conditions that did not require regular follow-up also decreased. People used to visit health facilities for minor complaints such as headache. However, most people preferred to use home-remedy instead of visiting a health facility during COVID-19.

*“After COVID-19 was reported the maximum number of patients we see per day was down to five. When we ask people why they are not coming, they tell as they are taking traditional medicines like garlic and garden cress.”*

**Effect on healthcare providers**

Participants indicated that fear among health professionals was common due to limited supply of PPE and small examination rooms to ensure social distancing. In addition, health professionals had limited information about prevention and management of COVID-19, and they didn’t have formal training. Rather, they were exposed to national and international media which explained the severity of the disease. This created fear, frustration, and anxiety among the providers. One participant indicated the situation as follows:

*“We were equally scared of the community as they were scared of us. The community feared us, and we feared the community.”*

In addition, COVID-19 created extra workload on the health professionals since they had additional COVID-19 response activities including screening at the gate and working in isolation rooms in addition to the routine services. Health providers were also required to serve clients beyond their usual catchment area when adjunct health facilities were converted to COVID-19 treatment/isolation centers. This resulted in high client flow in adjunct health facilities which resulted in increased workload.

Our findings indicated that health providers were discriminated by the community as they were considered a source of infection. A participant explained the situation as follows:

“*The community wasn’t very accepting of us, they used to mistreat the health professionals. Some even referred health professionals as “The COVID-19s”. The community used to say “The COVID-19s are here” when we go for home visits. We had a hard time.*”

**Effect on health services provision**

Our findings indicate that COVID-19 affected the quality of health services. Overall, COVID-19 affected patient-physician communication since some providers preferred to give services quickly to reduce their exposure. As a result, physical examination, and counseling services were compromised. One participant explained this as follows:

*“COVID-19 affected the service. I say this because we were limiting our contact hour with the clients. Immunization is a service where you provide vaccine, schedule next appointment, monitor growth, provide counselling about side effects, counsel about infection prevention and so on. This means it needs a lot of time to cover everything, but we were limiting our contact hour to prevent the infection, so all these services were compromised.”*

Some participants reported that the quality of service given for ART patients has been compromised. This is because newly diagnosed human immunodeficiency virus (HIV) patients used to be given treatment after repeated counselling with service providers and case manager to ensure their readiness and ability to sustain lifelong treatment. However, to minimize contact, new patients were prescribed medication on the same day they were diagnosed without such careful assessment of their readiness.

*“I have been working on ART unit for long and I can tell you that the quality of ART service has been compromised. We are giving ART medication for new HIV patients without checking whether they have accepted it or not. There are patients who need time to decide what they want to do, and previously we used to talk to them, and the case manager used to have repeated discussion with them before we put them on medication. But now we put them on medication on the same day they are diagnosed.”*

There was also a gap in monitoring viral load because of delay in laboratory test. Thus, patients stayed on medication that had to be changed since treatment regimen is based on viral load.

*“The other challenge that I want to tell you is related with viral load testing. We don’t know where our samples are. We get viral load test done at Ethiopia Public Health Institute but during the pandemic more focus was given for COVID sample, so we were getting incomplete results for the samples that we have sent. We even collected sample from our clients for a second round to get the viral load result. There was more than 6 months delay to get the result for viral load. This was the other main challenge that COVID posed on ART service.”*

Different investigations including organ function testing was also compromised because of a shortage of reagents. As a result, providers relied on signs and symptoms reported by the patients rather than laboratory tests to follow their patients.

**Theme 2: Adaptation strategies to sustain provision of essential healthcare services**

**Designating facilities as COVID-19 isolation and treatment centers**

As part of the pandemic response, one or two health centers from each sub-city were designated as COVID-19 isolation and/or treatment centers. This meant that non-COVID-19 cases and regular clients must go to alternate health centers. The selection of the health center as isolation and/or treatment center was based on experience of the health centers to manage outbreaks that have happened before. In addition, they selected health centers where the patient flow is low.

Challenge with this adaptation strategy was overcrowding of adjunct health facilities and burn out of healthcare providers as they were serving people from outside of their catchment areas where the health centers have been assigned as COVID-19 center.

**Prolonged prescription**

Participants explained that prolonged prescription of medication was the primary alternative mode of care that health centers used to sustain provision of different services. Health facilities started implementing prolonged prescriptions once direction was given by Ministry of Health, which was around 1 to 2 months after COVID-19 was first reported in Ethiopia. Prolonged prescription was implemented in all heath centers primarily for clients with follow-up such as RVI, TB and NCD patients. The period of prescription varied across different services. For instance, new RVI patients were given 3 months of supply during COVID. This, however, was not the practice before COVID-19 where new ART patients were given medication for two weeks in two rounds, then monthly medication followed by three months medication and finally six months medication by appointment spacing model designed for stable patients regularly checking their adherence and status.

*“We were given direction to use prolonged prescription. Therefore, what we did was, we took out all the patients’ card and we started calling our clients so that they can come and take their medication. We were giving them supply that would be enough for 3 months if they are new and 6 months for the rest of our clients.”*

Regarding TB services, health facilities previously followed directly observed therapy (DOTs) during the first two months of intensive treatment, during which time clients were expected to attend the health center daily. However, during the pandemic this modality was changed and clients in the intensive phase were given either a week or two weeks of supply depending on their condition. Likewise, during the second (continuation) phase of TB management clients normally come weekly for treatment. During the pandemic, this was changed so that clients were getting a monthly supply during this phase.

Similarly, NCD clients were given 3-6 months of supply to minimize frequency of contact. With this modality, health care providers were monitoring adherence of clients to their medication through phone calls, self-report during follow-up appointments and through viral load testing for ART clients.

The main reported challenges related with prolonged prescription include poor monitoring of adherence and poor adherence among clients resulting in treatment failure and complication of different cases. Prolonged prescription has also resulted in shortage of medication at the health center. Health facilities prescribe three months’ supply for NCD clients but there was shortage of supply at the health centers and clients were only able to purchase one-month supply from other governmental pharmacy.

**Community based health service**

The other mitigation strategy implemented by the health centers as explained by the participants was community-based provision of different services through primary health care team. This team is comprised of different service providers from different units including health extension workers (HEW). Though this system was in place before COVID-19, it was strengthened during the pandemic.

Some of the services provided by the team as explained by the participants included screening for NCD and TB cases, tracing loss to follow-up, provision of health education, distribution of Iron Folic Acid supplementation for pregnant women and vitamin A supplementation for children. This was captured as follows:

*“There is what we call primary health care team, and this team goes out to the community 4 days in a week. During community visit, health care provider in the team in collaboration with the HEWs create awareness about COVID-19 and other medical conditions. There is also provision of medication and family planning service for those who can’t come to health facility”*

Participants explained community-based distribution of medication was used for ART clients through a system called “Community ART Group (CAG)”. This system was in place before COVID-19, but it was strengthened during the pandemic through collaboration with non-governmental organizations. With this system, medications were distributed at community level either at school or church. This approach is captured as follows:

*“Using the community ART group, we (providers) arrange the community in a group of 5 or 6 then we go to the community and distribute the medication for the group. We were working in collaboration with non-governmental organization (NGO), and they allocated a car for us to deliver the service for the community.”*

**Prioritization**

Participants explained that health facilities prioritized certain services with the aim of freeing up the space and resource for the provision of other essential healthcare services. There were facilities that suspended services such as, medical service for a driving license, cervical cancer screening, minor surgeries such as circumcision. However, this only lasted for the first two to three months of the pandemic, and they resumed providing the service by taking the necessary precautions. One participant explained this as:

*“…..In addition, we suspended services like circumcision service and cervical screening service since they were not urgent. Service for general medical checkup was also suspended at first but then we started providing it because we believed we can sustain the provision by taking the necessary precaution. Now every service is being given.”*

Participants also explained that prioritization of clients was in place. All facilities prioritized clients who were believed to have high risk of getting seriously ill from COVID-19. These included patients with underlying medical conditions such as ART and NCD patients, pregnant women, and children. These clients were given priority to minimize waiting time to access services, thereby reducing their duration of stay at the HC. This is captured in the following quote:

*“The medical card of ART patients is kept in the case manager office, so they don’t have to wait in line to get their card. Thus, clients are able go straight to the case manager office and get the service in ART clinic which is located right next to the case manager office. This means they immediately get the service and leave.”*

The other strategy implemented by the health facilities to prioritize clients coming for ART services was through changes in work-hour arrangements. Providers started working early in the morning, during lunchtime and during Saturdays to provide ART service and to minimize crowding.

**Ensuring availability of resources**

Health centers took different measures to ensure adequate workforce and supply to sustain essential healthcare services. In terms of workforce, no service provider was allowed to take annual leave except for those who were high risk. These included service providers who were pregnant, elderly or who had comorbidities. In addition, providers on study leave were asked to return to work.

In terms of supplies, health centers used to borrow supplies from one another when they faced shortages. This includes supplies needed for essential services as well as personal protective equipment (PPE). Health facilities also reshuffled budgets such as shifting budget allocated for training to purchase PPE.

**Support**

According to our findings, different governmental and non-governmental organizations provided support for health facilities. Non-governmental organizations supported facilities by providing vehicles for CAG services, and mobile airtime cards used to trace clients and remind upcoming appointments. In addition, they provided logistic support such as masks, sanitizers, soap, and tents.

Federal and governmental offices also provided support for health centers by providing o masks and sanitizer. However, participants explained that the support from sub-city in terms of supportive supervision to ensure sustained provision of essential healthcare services reduced significantly.

*“Higher offices should have their own contribution to ensuring that services are being provided properly. There should have been supervision to ensure that services are being provided properly and that the community is not inconvenienced but no one came from higher offices to supervise because of fear of contracting an infection when they come to health center. People might be scared, and it is understandable, however, they should have monitored whether essential services were being provided properly or not.”*

*“We (personnel at sub-city) did not do that much monitoring and supervision. The professionals here at the sub-city did not want to go to health centers to monitor or supervise since there was frustration that resulted from fear of being infected from going to health facilities. So, one of the drawbacks of COVID-19 was the visible collapse of service deliveries, because of the poor support and supervision of health services.”*

**Infection prevention and control (IPC) measures**

Initiative of renovating facilities for infection prevention including securing water supplies was done by sub cities by following strict application of guidelines.

Regarding providers’ IPC implementation practice, most healthcare providers explained that there was a stricter adherence to infection prevention activities like wearing mask and washing or sanitizing hands. However, the use of masks and sanitizers has loosened over time.

“*We use sanitizer and mask, but we hug and kiss. we also used to eat lunch separately by keeping distance but now we are eating in one dish, which was the custom before COVID-19, Everything is getting back to normal.”*

As for IPC measures taken for and by patients, initially, temperature screening at the health facility entrance, isolation of clients with symptom of COVID-19, social distancing in waiting areas, mandatory mask use and hand washing was enforced. There was also repurposing of rooms for better ventilation if the previously used rooms were too small. Although the IPC measures were strictly implemented during the first few months, practices have become lapsed as indicated by our participants.

Participants explained the different challenges they have faced related with IPC measures. The challenges cited include, lack of well-equipped isolation room, shortage of PPE including mask, sanitizer, glove, and water especially during the first 2-3 months resulting in frustration among service providers.

*“We had shortage of PPE and the budget that was allocated for health centers was very low this year. It was even lower than last year’s budget, so we were challenged to equip the health center with the necessary logistics. In addition, the price of materials has gone up this year. A single surgical mask that used to cost around 3 birr now costs around 9 or 10 birr. So, we struggled to purchase a mask. We used to give providers one mask for a weak and half litter sanitizer for a month. This has made the providers feel uncomfortable.”*

Another challenge related with IPC measures were shortage of isolation room and poor infrastructure of health facilities to ensure safe delivery of different services and negligence from the community/clients towards prevention strategies. There was also a challenge related with sustaining the implementation of different prevention strategies including screening at the gate, ensuring clients wash their hands and ensuring social distancing at the health centers.

Poor preparedness was one of the reasons identified by participants that has resulted in shortage of supply.

*"We could have been more prepared. There was negligence starting from higher health office, otherwise we would not have shortage of logistic. If we thought about this earlier, we would not have such shortage. If you go to the pharmaceutical supply agency, they will tell you that they don’t have mask even though they have, and this shows you that they were not ready and they didn’t have adequate stock."*

**Discussion**

This study explored the health system response to maintain provision of essential health care services in Addis Ababa, Ethiopia during the COVID-19 pandemic. We found that COVID-19 affected delivery of essential health service in various ways during the early stages of the pandemic. The pertinent effects were perceived decline in health facility utilization, fear of infection among healthcare providers, stigma towards healthcare providers and a general decrease in quality of services. Overall, health system actors made efforts to sustain services while responding to the pandemic through providing supplies, and enacting changes in the service delivery modality. The most significant changes in the service delivery included repurposing health centers and prolonged prescription. The primary challenges encountered were burnout of health workforce and shortage of PPE.

COVID-19 has challenged the health service delivery and resulted in a reduction of healthcare services utilization globally (3–10,16,23,26). Our findings also indicated a perceived decrease of health facility visits to utilize essential health care services. The primary reason for this was perceived fear of infection among the community. This is supported by similar studies in other countries that emphasize the effect of fear on service utilization (27–31). Fear was greatest in the earlier phase of the pandemic, where less was understood about the pandemic. Fear among the community might have been exacerbated by the media’s portrayal of the pandemic(32). In particular, the public was consistently advised that people with comorbidity were at higher risk of contracting infection. Such messages might have inadvertently discouraged people with co-morbidities from visiting health facilities for essential healthcare services. In addition, rumors in the community about quarantine/isolation might have impacted clients’ decision of not visiting health facility for essential health services.

Our finding indicated fear was common among health care providers as well. This is supported by other studies that indicated fear to be the most common psychological reaction among health care providers who continue to provide healthcare services during the COVID-19 pandemic (30,33–35). Different reasons contributed to health care provider’s fear, one of the most common reasons was lack of personal protective equipment especially at the beginning of the pandemic. Shortage of PPE is also reported by other similar studies as a major challenge contributing to health care provider’s fear (32,36,37).

Other reasons for fear include fear of stigma and fear of carrying the virus back home and infecting loved ones (33,38). Such psychological distress coupled with high workload demand among health care providers could result in burnout and compromise quality of service. Hence it is important for governments and policy makers to consider and allocate funding to promote and provide psychosocial support for health care providers during a pandemic.

Health system actors made effort to sustain the use of essential health care services through enacting changes in the service delivery. The primary change was the use of multi-month dispensing of medication for up to six months for people who may be at increased risk of COVID-19, such as people living with comorbidity. Even though this modality can minimize the risk of COVID-19 infection, there is a chance that some patients might suffer from complications because of limited follow-up of the diseases. In addition, there was no mechanism to monitor medication adherence other than self-report, where the validity can be affected by the recall period. Since medication adherence plays an important role in optimizing health outcomes, a mechanism to monitor adherence should be put in place instead of relying on self-report when using prolonged prescription (39).

Designating health centers as COVID-19 treatment/isolation was another response by the health system. In this study, healthcare providers explain that though this approach appears to have been effective in ensuring the sustained provision of services, it has increased their workload. This is because providers in non-COVID-19 treatment/isolation centers were serving clients outside their catchment population. In addition, they were providing other COVID-19 prevention activities such as screening, isolating, and engaging in community-based information dissemination, which resulted in burnout. Similarly, burnout among health workforce has been identified as a challenge in other studies (13,34,40,41). Burnout among health care providers has been associated with poor quality of care (42). Thus, it is crucial for organizational leaders and policy makers to mitigate and reduce burnout among healthcare providers during pandemic.

**Strengths and limitations**

Our current study has limitations that need to be acknowledged. This study was conducted only in Addis Ababa city; therefore, the findings may not apply to other regions. The other limitation is that it lacks the community/patient perspective. The strength of this study is it involved stakeholders starting from the ministry level down to the frontline health workers, which provides a comprehensive view of experience. In addition, we interviewed service providers from different contact points, which enabled us to capture the mitigation strategies at different contact points.

**Conclusion and recommendations**

The experiences of healthcare professionals in Addis Ababa during the initial phase of the pandemic highlights reports of issues relating to the impact of COVID-19 on the provision of essential health care services, as well as the attempts undertaken to sustain health care provision and associated challenges. Even though participants’ perspectives are embedded in a specific standpoint during a specific period, their experiences resemble those faced by health systems in other settings during the pandemic.

The COVID-19 presented multifaceted challenges to the health system: from fear among health care provider and perceived reduction in health care utilization, to decreased quality in health service provision. While health system actors made concerted efforts to respond to the pandemic while sustaining provision of essential healthcare services through various adaptation strategies, there were inherent shortcomings during application. Therefore, it is crucial for health systems to carefully evaluate adaptation strategies as they have their own setbacks that should be addressed carefully.

**List of Abbreviation**

ANC: Antenatal Care

ART: Antiretroviral Therapy

CAG: Community Antiretroviral Therapy Group

DOT: Directly Observed Therapy

FMOH: Federal Ministry of Health

HC: Health Center

HEW: Health Extension Worker

HIV: Human Immunodeficiency Virus

IPC: Infection Prevention Control

NCD: Non-Communicable Diseases

NGO: Non-Governmental Organization

PHCU: Primary Health Care Unit

PPE: Personal Protective Equipment

TB: Tuberculosis

**Declarations**

**Ethics approval and consent to participate**

Ethical clearance was obtained from the Research Ethical Committee of School of Public Health, Addis Ababa University (reference number 070/20SPH) and University of Liverpool (reference number 8049). In addition, permission was obtained from relevant federal and regional health offices. Written informed consent was obtained from the participants after the necessary explanation about the purpose, procedures, benefits, and risk of the study had been made. The respondent’s right to refuse few or all the questions was respected at all times. In addition, privacy of participants and confidentiality of the information obtained was always maintained.

**Consent for Publication**

Not Applicable

**Availability of data and materials**

The datasets generated and/or analyzed during the current study are available from the corresponding author on reasonable request.

**Competing Interests**

All authors declare that they have no competing interests.

**Funding**

This research was supported by the Global Challenges Research Fund (GCRF) One Health Regional Network for the Horn of Africa (HORN) Project, from UK Research and Innovation (UKRI) and Biotechnology and Biological Sciences Research Council (BBSRC) (project number BB/P027954/1). The funders had no role in study design, data collection and analysis, decision to publish, or preparation of the manuscript.

**Author’s contribution**

Conceived and designed the study: FB, EH, BT, AA, ML. Data collection: FB, EH, BT, AA, ML. Data transcription and coding: FB, EH, BT, AA, ML. Data analysis: FB, EH, BT, AA, ML. original draft write-up: FB, EH, BT, AA, ML. Critical review of the manuscript: JP, SM, MK. Editing the manuscript: FB, EH, BT, AA, ML. All authors have read and approved the manuscript.

**Acknowledgments**

We would like to thank the HORN regional network for the Horn of Africa program for making this study possible by providing both financial and technical support and platform. We would like to extend our gratitude to our mentors for guiding the conduction of this study. We also want to thank the participants of this study without whom the study would not have been a reality.

**Reference**

1. Ethiopian Public Health Institute. COVID-19 Pandemic Preparedness and Response in Ethiopia weekly Bulletin No. 48, March 22-28, 202. [Internet]. Available from: [www.ephi.gov.et](http://www.ephi.gov.et).

2. Centers for Disease Control and Prevention. Maintaining essential health services during COVID-19 in low resource, non-US settings.

3. Abebe W, Worku A, Moges T, Tekle N, Amogne W, Haile T, Mekonen D, Habtamu A, Deressa W. Trends of follow-up clinic visits and admissions three-months before and during COVID-19 pandemic at Tikur Anbessa specialized hospital, Addis Ababa, Ethiopia: an interrupted time series analysis. BMC Health Services Research. 2021 Dec;21:1-0

4. Moynihan R, Sanders S, Michaleff ZA, Scott AM, Clark J, To EJ, Jones M, Kitchener E, Fox M, Johansson M, Lang E. Impact of COVID-19 pandemic on utilisation of healthcare services: a systematic review. BMJ open. 2021 Mar 1;11(3):e045343.

5. Haldane V, De Foo C, Abdalla SM, Jung AS, Tan M, Wu S, Chua A, Verma M, Shrestha P, Singh S, Perez T. Health systems resilience in managing the COVID-19 pandemic: lessons from 28 countries. Nature Medicine. 2021 Jun;27(6):964-80.

6. Desta AA, Woldearegay TW, Gebremeskel E, Alemayehu M, Getachew T, Gebregzabiher G, et al. Impacts of COVID-19 on essential health services in Tigray, Northern Ethiopia: A prepost study. PLoS One. 2021 Aug 1;16(8 August).

7. Workicho A, Kershaw MJ, Berhanu L, Kebede M, Kennedy E. Essential health and nutrition service provision during the COVID-19 pandemic: lessons from select Ethiopian Woredas. Current Developments in Nutrition. 2021 Apr;5(4): nzab024.

8. Dandena F, Teklewold B, Anteneh D. Impact of COVID-19 and mitigation plans on essential health services: institutional experience of a hospital in Ethiopia. BMC Health Serv Res. 2021 Dec 1;21(1).

9. Abdela SG, Berhanu AB, Ferede LM, van Griensven J. Essential healthcare services in the face of COVID-19 prevention: Experiences from a referral hospital in Ethiopia. American Journal of Tropical Medicine and Hygiene. 2020 Sep 1;103(3):1198–200.

10. World Health Organization. Pulse survey on continuity of essential health services during the COVID-19 pandemic: interim report, 27 August 2020. World Health Organization; 2020.

11. Alhalaseh YN, Elshabrawy HA, Erashdi M, Shahait M, Abu-Humdan AM, Al-Hussaini M. Allocation of the “Already” Limited Medical Resources Amid the COVID-19 Pandemic, an Iterative Ethical Encounter Including Suggested Solutions From a Real Life Encounter. Vol. 7, Frontiers in Medicine. Frontiers Media S.A.; 2021.

12. Emanuel EJ, Upshur R, Thome B, Parker M, Glickman A, Zhang C, et al. Fair Allocation of Scarce Medical Resources in the Time of Covid-19. 2020.

13. Stone KW, Kintziger KW, Jagger MA, Horney JA. Public health workforce burnout in the covid-19 response in the u.s. Int J Environ Res Public Health. 2021 Apr 2;18(8).

14. Barasa E, Kazungu J, Orangi S, Kabia E, Ogero M, Kasera K. Indirect health effects of the COVID-19 pandemic in Kenya: a mixed methods assessment. BMC Health Serv Res. 2021 Dec 1;21(1).

15. World Health Organization. Second round of the national pulse survey on continuity of essential health services during the COVID-19 pandemic: January-March 2021: interim report, 22 April 2021. World Health Organization; 2021.

16. Ayele W, Biruk E, Kifle A, Habtamu T, Taye G, Wondarad Y. Patterns of essential health services utilization and routine health information management during Covid-19 pandemic at primary health service delivery point Addis Ababa, Ethiopia.

17. World Health Organization. Maintaining essential health services: operational guidance for the COVID-19 context: interim guidance, 1 June 2020. World Health Organization; 2020.

18. Inzaule SC, Ondoa P, Loembe MM, Tebeje YK, Ouma AE, Nkengasong JN. COVID-19 and indirect health implications in Africa: Impact, mitigation measures, and lessons learned for improved disease control. PLoS Medicine. 2021 Jun 23;18(6):e1003666.

19. Charles J. Essential Package of Health Services Photo Credit: Ethiopia Private Sector Health Program (PSHP) Avenir Health | Broad Branch Associates | Development Alternatives Inc. (DAI) | | Johns Hopkins Bloomberg School of Public Health (JHSPH) | Results for Development Institute (R4D) | RTI International | Training Resources Group. 2015.

20. El Bcheraoui C, Weishaar H, Pozo-Martin F, Hanefeld J. Assessing COVID-19 through the lens of health systems’ preparedness: time for a change. Globalization and Health. 2020 Dec;16:1-5.

21. FMOH E. National comprehensive Covid19 management handbook. Ethiopian Federal Ministry of Health. 2020 Apr.

22. Ababa A. Essential Health Services Package of Ethiopia.

23. Lieneck C, Herzog B, Krips R. Analysis of facilitators and barriers to the delivery of routine care during the COVID-19 global pandemic: A systematic review. Vol. 9, Healthcare (Switzerland). MDPI AG; 2021.

24. Federal Democratic Republic of Ethiopia Ministry of Health. Health Sector Development Program IV 2010/11-2014/15. Ministry of Health Addis Ababa. 2010 Oct.

25. Braun V, Clarke V. Using thematic analysis in psychology. Qualitative research in psychology. 2006 Jan 1;3(2):77-101.

26. Tumwesigye NM, Denis O, Kaakyo M, Biribawa C. Effects of the COVID-19 Pandemic on Health Services and Mitigation Measures in Uganda [Internet]. Vol. 416, CGD Working Paper. 2003. Available from: www.cgdev.orgwww.cgdev.org

27. Xiao H, Dai X, Wagenaar BH, Liu F, Augusto O, Guo Y, et al. The impact of the COVID-19 pandemic on health services utilization in China: Time-series analyses for 2016–2020. Lancet Reg Health West Pac. 2021 Apr 1;9.

28. Modesti PA, Wang J, Damasceno A, Agyemang C, van Bortel L, Persu A, et al. Indirect implications of COVID-19 prevention strategies on non-communicable diseases. BMC Med. 2020 Aug 14;18(1).

29. Giamello JD, Abram S, Bernardi S, Lauria G. The emergency department in the COVID-19 era. Who are we missing? Vol. 27, European Journal of Emergency Medicine. Lippincott Williams and Wilkins; 2020. p. 305–6.

30. Singh DR, Sunuwar DR, Shah SK, Karki K, Sah LK, Adhikari B, et al. Impact of COVID-19 on health services utilization in Province-2 of Nepal: a qualitative study among community members and stakeholders. BMC Health Serv Res. 2021 Dec 1;21(1).

31. Zhao J, Li H, Kung D, Fisher M, Shen Y, Liu R. Impact of the COVID-19 Epidemic on Stroke Care and Potential Solutions. Stroke. 2020;1996–2001.

32. Billings J, Ching BCF, Gkofa V, Greene T, Bloomfield M. Experiences of frontline healthcare workers and their views about support during COVID-19 and previous pandemics: a systematic review and qualitative meta-synthesis. BMC Health Serv Res. 2021 Dec 1;21(1).

33. Juan Y, Yuanyuan C, Qiuxiang Y, Cong L, Xiaofeng L, Yundong Z, et al. Psychological distress surveillance and related impact analysis of hospital staff during the COVID-19 epidemic in Chongqing, China. Compr Psychiatry. 2020 Nov 1;103.

34. Thatrimontrichai A, Weber DJ, Apisarnthanarak A. Mental health among healthcare personnel during COVID-19 in Asia: A systematic review. Vol. 120, Journal of the Formosan Medical Association. Elsevier B.V.; 2021. p. 1296–304.

35. Ching SM, Ng KY, Lee KW, Yee A, Lim PY, Ranita H, et al. Psychological distress among healthcare providers during COVID-19 in Asia: Systematic review and meta-analysis. Vol. 16, PLoS ONE. Public Library of Science; 2021.

36. Razu SR, Yasmin T, Arif TB, Islam MS, Islam SMS, Gesesew HA, et al. Challenges Faced by Healthcare Professionals During the COVID-19 Pandemic: A Qualitative Inquiry From Bangladesh. Front Public Health. 2021 Aug 10;9.

37. Deressa W, Worku A, Abebe W, Gizaw M, Amogne W. Availability and use of personal protective equipment and satisfaction of healthcare professionals during COVID-19 pandemic in Addis Ababa, Ethiopia. Archives of Public Health. 2021 Dec 1;79(1).

38. Grover S, Singh P, Sahoo S, Mehra A. Stigma related to COVID-19 infection: Are the Health Care Workers stigmatizing their own colleagues? Vol. 53, Asian Journal of Psychiatry. Elsevier B.V.; 2020.

39. Stirratt MJ, Dunbar-Jacob J, Crane HM, Simoni JM, Czajkowski S, Hilliard ME, et al. Self-report measures of medication adherence behavior: recommendations on optimal use. Vol. 5, Translational Behavioral Medicine. Springer New York LLC; 2015. p. 470–82.

40. Impact on Essential Health Services The Independent Panel for Pandemic Preparedness and Response The Secretariat for the Independent Panel for Pandemic Preparedness and Response. 2021.

41. Maunder RG, Heeney ND, Strudwick G, Shin HD, O’Neill B, Young N, et al. Burnout in Hospital-Based Healthcare Workers during COVID-19 [Internet]. 2021 Oct. Available from: https://covid19-sciencetable.ca/sciencebrief/burnout-in-hospital-based-healthcare-workers-during-covid-19

42. Tawfik DS, Scheid A, Profit J, Shanafelt T, Trockel M, Adair KC, et al. Evidence relating health care provider burnout and quality of care a systematic review and meta-analysis. Vol. 171, Annals of Internal Medicine. American College of Physicians; 2019. p. 555–67.
